# Editorial: Gene Silencing and Editing Strategies for Neurodegenerative Diseases

**DOI:** 10.3389/fnins.2018.00425

**Published:** 2018-06-22

**Authors:** Clévio Nóbrega, Sandro Alves

**Affiliations:** ^1^Department of Biomedical Sciences and Medicine, University of Algarve, Faro, Portugal; ^2^Centre for Biomedical Research, University of Algarve, Faro, Portugal; ^3^Algarve Biomedical Center, University of Algarve, Faro, Portugal; ^4^Center for Neuroscience and Cell Biology, University of Coimbra, Coimbra, Portugal; ^5^Brainvectis Therapeutics, Paris, France

**Keywords:** neurodegenerative diseases, gene silencing, gene editing, neurodegeneration, therapies

The discovery of the RNA interference (RNAi) mechanism and the development of gene editing techniques such as TALENs or CRISPR/Cas9 opened a new avenue for the treatment and understanding of neurodegenerative diseases. If the main goal and most straightforward rationale is to use these systems to develop therapeutic options for neurodegenerative diseases, it is also clear that RNAi and editing strategies are valuable for research, as tools for the understanding of the molecular mechanisms implicated in these diseases or, alternatively, to develop research models for their study.

Recent studies have demonstrated that several biological networks implicated in the modulation of gene expression at transcriptional, post-transcriptional and epigenetic levels, are tightly regulated by multiple long non-coding RNAs (lncRNAs), some of them specifically expressed in the central nervous system (CNS). These lncRNAs may have an important role in the pathophysiology of neurodegenerative diseases. So far, deregulation of most lncRNAs is widely associated to the progression of neurodegenerative disorders like Alzheimer's (AD), Parkinson's (PD), and Huntington's (HD) diseases, as well as Amyotrophic Lateral Sclerosis (ALS). Quan et al. comprehensively discuss several lncRNAs-associated regulatory mechanisms, which may be involved in the pathophysiology of the above-mentioned diseases. They conclude that additional investigation of lncRNAs rolls has the potential to achieve increasing insights about their functions and therefore to better engineer therapeutic strategies based on lncRNAs modulation.

## Silencing and editing strategies as therapeutic approaches

Recently, the toxicity at the RNA level emerged as a new important player in the pathogenesis of HD and other Polyglutamine (polyQ) disorders. Several studies have demonstrated cell degeneration and dysfunction associated to the expression of untranslated transcripts with expanded CAG repeats. Therefore, the development of editing strategies provided the tools and opportunity to directly target the mutant gene, thus preventing the formation of toxic RNA and the consequent cascade of pathological events. In line with this idea, Dabrowska et al. developed a CRISPR/Cas9 strategy based in the precise excision of the CAG tract using derived fibroblasts from several HD patients. This strategy was successful in excising the repeats leading to a safe and specific abolition of huntingtin synthesis. These important results if translated to *in vivo* studies could become a therapeutic option in future clinical trials for HD patients.

Another gene silencing-based therapy is the use of antisense oligonucleotides delivered by calcium-phosphate-lipid nanoparticles. The goal is to lower mutant superoxide dismutase I (SOD1), that abnormally accumulates in ALS motor neurons. Thus, SOD1 lowering could be an effective therapeutic strategy. To circumvent the short half-life of ASOs and the fact that they cannot cross the blood-brain-barrier, Chen et al. propose calcium phosphate lipid coated nanoparticles-based delivery of ASOs targeting SOD1 in motor neurons, as an effective and safe delivery system to the CNS. The authors also report for the first time the distribution of nanoparticles in the brain and spinal cord of zebrafish, a powerful animal model to develop therapies for ALS and other neurological disorders.

## Silencing and editing strategies as research tool

### To understand the molecular mechanisms underlying neuronal events

Methamphetamine (METH) is a stimulant drug that induce toxicity in the CNS, especially in dopaminergic neurons. Du et al. explored the effect of METH in astrocyte-related neuroinflammation, using siRNAs to block the expression of Caspase-11 and toll-like receptor (TLR4) in primary cultures of mouse astrocytes. They found that siRNA inhibition of Caspase-11 reduce the expression of pro-inflammatory cytokines, whereas TLR4 inhibition decrease the METH-induced activation of NF-kB and Caspse-11. These results highlight the importance of both Caspase-11 and TLR4 in the neuroinflammation induced by METH.

Inflammation is induced upon cerebral ischemia, with an activation of microglia and their phenotype alteration to M1 or M2, depending on the microenvironment stimuli. The study by Cheon et al. use siRNA-based strategies to study the involvement of signal-regulating kinase 1 (ASK1) in microglia function and its alterations upon ischemic stroke. The results show that ASK1 inhibition suppressed the expression of M1-associated genes (pro-inflammatory mediators), whereas augmented M2-associated genes expression (anti-inflammatory mediators). The authors propose that ASK1 modulation might constitute a new strategy for repair, targeting the microglia phenotype after an ischemic stroke.

Cholesterol metabolism has been widely associated to several neurodegenerative diseases including Alzheimer's disease. Despite this, lipid modifications associated to the disease progression, as well as the link between altered cholesterol levels and AD still remains poorly understood. In Ayciriex et al. the authors promoted the silencing of the *Cyp46a1* gene (which encodes the cholesterol 24-hydroxylase) in the mouse hippocampus and performed both targeted and non-targeted approaches by liquid chromatography coupled to high resolution mass spectroscopy to better understand lipid modifications associated to AD-like degeneration. The authors show that the cholesterol 24-hydroxylase silencing, key enzyme in cholesterol metabolism deregulates lipid homeostasis in brain, thus allowing dissecting the role of cholesterol metabolism in AD and other neurodegenerative diseases.

Mitochondrial dysfunction underlies the pathogenesis of several neurodegenerative diseases, including PD. Ye et al. use the RNAi pathway to inhibit peroxisome proliferator-activated receptor γ coactivator-1alpha (PGC-1α) in a PD cellular model. PGC-1α is implicated in the regulation of mitochondrial biogenesis and oxidative capacity. The use of siRNAs reducing the levels of PGC-1α led to a reduction of mitochondrial membrane potential, intracellular ATP content and intracellular H_2_O_2_ generation, thus establishing PGC-1α as an important modulator of mitochondria function.

In summary, all these studies highlight the importance of gene silencing strategies to achieve a better understanding of biological cellular mechanistic pathways.

### To develop research models

The development of research models is an essential tool for studying the molecular and cellular events underlying a pathogenesis and for testing new therapeutic approaches. Induced pluripotent stem cells (iPSCs), became one of the most popular research models, providing the opportunity to have customized models for diseases and patients. Szlachcic et al. developed a study aiming the development of HD iPS cells, where huntingtin was silenced by shRNAs. The authors achieved a stable silencing of huntingtin in different HD iPSc lines, remaining active until the differentiation in neural stem cells. The authors used the developed model to study the effect of huntingtin silencing in different signaling pathways, providing evidences for usefulness of the model to study the molecular processes underlying HD pathogenesis.

All these studies highlight that gene silencing and editing tools are powerful tools for research, allowing the development of advanced therapies based in gene and cell delivery, and importantly allowing the modulation of cellular pathways and functions as well as to develop new models to be used for research.

## Author contributions

CN and SA contributed equally to this editorial, being both involved in the outline of the editorial, in its writing and scientific review.

### Conflict of interest statement

The authors declare that the research was conducted in the absence of any commercial or financial relationships that could be construed as a potential conflict of interest.

